# Food protein-induced enteropathy: a revision for the clinician

**DOI:** 10.3389/fped.2024.1417391

**Published:** 2024-09-10

**Authors:** Simona Barni, Francesca Mori, Luca Pecoraro, Francesca Saretta, Mattia Giovannini, Stefania Arasi, Lucia Liotti, Carla Mastrorilli, Angela Klain, Mariannita Gelsomino, Riccardo Castagnoli, Michele Miraglia del Giudice, Elio Novembre

**Affiliations:** ^1^ Allergy Unit, Meyer Children’s Hospital IRCCS, Florence, Italy; ^2^ Pediatric Unit, Department of Surgical Sciences, Dentistry, Gynecology and Pediatrics, University of Verona, Verona, Italy; ^3^ General Pediatrician, Azienda Sanitaria Universitaria Friuli Centrale, Udine, Italy; ^4^ Department of Health Sciences, University of Florence, Florence, Italy; ^5^ Translational Research in Pediatric Specialties Area, Division of Allergy, Bambino Gesù Children’s Hospital, IRCCS, Rome, Italy; ^6^ Pediatric Unit, Department of Mother and Child Health, Salesi Children’s Hospital, Ancona, Italy; ^7^ Pediatric and Emergency Department, Pediatric Hospital Giovanni XXIII, AOU Policlinic of Bari, Bari, Italy; ^8^ Department of Woman, Child and General and Specialized Surgery, University of Campania Luigi Vanvitelli, Naples, Italy; ^9^ Department of Life Sciences and Public Health, Pediatric Allergy Unit, University Foundation Policlinico Gemelli IRCCS, Catholic University of the Sacred Heart, Rome, Italy; ^10^ Pediatric Clinic, Fondazione IRCCS Policlinico San Matteo, Pavia, Italy

**Keywords:** children, enteropathy, diarrhea, food allergy, non-IgE mediated, vomiting

## Abstract

Food protein-induced enteropathy (FPE) belongs to non-IgE gastrointestinal mediated food allergies. FPE is a syndrome characterized by diarrhea, weight loss and failure to thrive in young infants. Cow milk is the culprit food that most frequently causes FPE. The prevalence of FPE has not been fully estimated, but it is relatively rare. The diagnosis is based on the clinical manifestations and histological findings through colonoscopy. Laboratory tests are somewhat helpful in the diagnosis, although there are no disease-specific findings. Allergy testing for food specific IgE is not routinely recommended. The cornerstone of the management of FPE is the removal of culprit food from the diet. FPE is usually a transient condition that resolves in most cases by 1–2 years of life. This review addresses the latest findings on FPE, including a practical guide to assist pediatricians treating children with FPE.

## 1 Introduction

Along with food protein induced allergic proctocolitis (FPIAP) and food protein induced enterocolitis syndrome (FPIES), food protein induced enteropathy (FPE) belongs to non-IgE gastrointestinal mediated food allergies (non-IgE-GI-FA) (1). Moreover, FPE is one of the causes of protein losing enteropathy along with eosinophilic gastroenteritis (2).

FPE is a syndrome characterized by diarrhea, weight loss and failure to thrive (FTT) in young infants, as described for the first time in the 1960s by different authors (3–6). Malabsorption affecting fats and carbohydrates as well as other nutrients can be evidenced through laboratory tests. It is not primarily due to deficiency of digestion but to changes in the structure of the intestinal mucosa (7). The culprit food most frequently causing FPE is cow milk (CM), although other foods have also been reported in the literature (8, 9).

## 2 Epidemiology

The prevalence of FPE has not been fully estimated, but it is relatively rare. After its recognition in the 1960s, an average rate of 3 patients per year with CM-FPE was observed in Finnish hospitals from 1962 to 1972. In the following years, (1973–1977) such rate raised to 5 patients per year, who, however, presented a milder phenotype with normal jejunal structure and no malabsorption syndrome (7, 10). Since then, this type of severe jejunal mucosal damage caused by CM has almost completely disappeared from Finland. A similar trend was described in Spain from 1980 to 1987 (11). Possible explanations for this gradual reduction in the incidence of CM-induced FPE may be due to several changes concerning feeding practices that have been implemented in Finland since the 1970s. Indeed, before the 1970s, breastfeeding was shorter in duration than after the 1970s (10). At the same time, homemade cow milk-based mixtures were replaced first by non-adapted and later by adapted formulas. The protein content of the formulas was reduced during the 1980s. Changes in nutritional practices, together with other quick changes in society, may have affected the microbial flora of the gut in infants, with a possible impact on the occurrence of atopic diseases in general (12, 13) and of this specific syndrome (7).

Patients with FPE have concomitant atopic diseases in 40%–50% of cases (1). Concomitant severe atopic dermatitis may lead to severe hypoproteinemia due to protein loss, not only from the gut but also and especially from the skin (14, 15).

FPE with a more protracted clinical course has been described in patients with Down syndrome (10, 16, 17). This may be due to an intrinsic defect of the immune system (18) that includes altered secretion of α-TNF and IL-10 (19, 20).

## 3 Pathophysiology

In FPE, the infiltration of food-specific (particularly CM) T-cells seems to be the reason behind the structural damage of the digestive mucosa, causing malabsorption (7, 21). Substantial evidence supports cytotoxic CD8+ T cells playing a central role in the pathogenesis of FPE. Such subset of T lymphocytes can directly kill target cells, including those in the intestinal mucosa, resulting in the typical symptoms of FPE, e.g., chronic diarrhea and malabsorption (22, 23). From 50 to 100% of patients affected by severe FPE showed an increased density of intraepithelial cells γδ-TCR+, similarly to celiac disease (CD) and autoimmune enteropathy (7, 24, 25). Koikkonen et al. (26) studied 15 children with a confirmed diagnosis of CM protein-sensitive enteropathy (CMSE), 12 with suspected cases of CMSE, 11 with CD, and 12 control children. CMSE was confirmed through a blind OFC. Common observations among children with CMSE included visible lymph nodular hyperplasia of the duodenal bulb and lymphoid follicles in biopsy samples, without villous atrophy. Patients with confirmed CMSE displayed notably higher densities of intraepithelial T cells – particularly γδ+ cells – compared to the control group, but lower than those observed in patients with CD.

Increased numbers of cytotoxic intraepithelial lymphocytes expressing T-cell-restricted intracellular antigen have been found in biopsy specimens from infants as well as school-age children (23, 27, 28). Moreover, a study conducted by Carroccio et al. (29) on adults found out that 86% of patients had malabsorption symptoms during CM-OFC, while 60% of patients had intraepithelial lymphocytes in the duodenal mucosa, showing cow milk protein hypersensitivity. A similar pathogenetic pattern can be found in patients suffering from non-celiac gluten sensitivity (30).

Activation of cytotoxic duodenal intraepithelial lymphocytes (IELs) is also confirmed by analyzing the expression of cytotoxic granule components such as perforin and granzyme A and B 91 (23, 31). Such IELs correlates with Fas ligand concentration, suggesting a role of Fas-mediated apoptosis in the pathogenesis (32).

In addition to lymphocytic infiltration, eosinophilic and mast cell infiltration and degranulation have been found in biopsies from infant with CM-FPE with increased level of histamine and extracellular major basic protein (MBP). Moreover, the deposition of MPB-positive correlates with the severity of villous atrophy (33, 34).

According to some immunohistochemical studies of the mucosal biopsies, humoral changes could be related to a nonspecific increase in mucosal IgA, IgG and IgM, with inconsistent increase on IgE (9, 35).

As per the cytokine pattern, the stimulation with CM protein *in vitro* produced higher levels of interferon (IFN) gamma (γ) and interleukin (IL) 4 from the jejunal mucosa in patients affected by CM-FPE than in the control patients. Also, the amount of IFN-γ- secreting cells is 10 time higher than IL-4- secreting cells. Finally, IL-10-secreting cells were reduced (36).

## 4 Clinical manifestations

In FPE, patients usually develop symptoms resembling those of CD and appearing in young infants (<9 months of age, usually in the first 1–2 months) whose diet does not include cereals yet.

Among such symptoms often are chronic diarrhea and malabsorption features like steatorrhea and FTT, their weight being more affected than their height. Vomiting is also frequently reported. In most cases, symptoms onset is reported shortly – within a few weeks – after the introduction of CM in the infant's diet (7, 10, 16, 37–39).

The onset tends to be gradual, sometimes imitating acute gastroenteritis with transient emesis complicated by protracted diarrhea. As these two conditions may overlap, distinguishing FPE from post-enteritis-induced lactose intolerance might be difficult (40).

Acute damage to the small intestine due to gastroenteritis might predispose infants to FPE; another hypothesis is that it may reveal an underlying hypersensitivity to food proteins (41–44).

In the majority of infants, diarrhea disappears within a week since the elimination diet of the culprit food has started (7), although some infants may require prolonged parenteral nutrition (16). Unlike CD, extra-digestive symptoms such as dermatitis herpetiformis are usually absent in FPE.

## 5 Trigger foods

CM is known to be the food trigger that most frequently causes food allergy in children (45); therefore, it is not surprising that even in non-IgE-GI-FA, which accounts for 40%–50% of food allergies (46–48), CM is the allergen most frequently causing symptoms including in children affected by FPE.

Previous studies conducted to evaluate trigger foods in patients with FPE report that CM is the main culprit food (10, 11, 16, 49). In a study conducted in Finland on 54 patients with CM-FPE (16), co-allergy with soy and with wheat is reported, respectively, in 11% and 37% of the patients tested. Eggs and banana were other allergens reported in 4% of patients and meat in 2% of patients.

## 6 Diagnosis

The diagnosis of non-IgE-GI-FA is based on the clinical manifestations except for FPE, which usually requires histological confirmation. There are no validated diagnostic criteria for FPE, although some elements routinely used in clinical practice are considered to support the diagnosis (39, 50, 51):

1. FPE usually occurs in young infants (<9 months), although it may present in older children.
2. Repeated exposure to trigger food elicits typical gastrointestinal symptoms, mainly vomiting and/or diarrhea, within 40–72 h.
3. Histological confirmation of the diagnosis in symptomatic children through small bowel biopsy shows villous injury, crypt hyperplasia and inflammation.
4. Elimination diet of the culprit food results in clinical (within 1–4 weeks) and histological remission (although complete healing of villous injury may take several months).
5. Other possible causes of vomiting and FTT must be excluded.

## 7 Oral food challenge (OFC)

The OFC is the gold standard to confirm the diagnosis of FPE after the resolution of symptoms during an elimination diet. Reintroduction of the trigger food elicits typical gastrointestinal symptoms – mainly vomiting and/or diarrhea – within 40–72 h (51). Reintroduction of culprit food 4–8 weeks after its elimination can usually be performed at home (51), if allergy tests are negative and if there is no history of previous severe symptoms (52).

## 8 Investigations

### 8.1 Laboratory tests

Laboratory tests are somewhat helpful in the diagnosis of non-IgE-GI-FA, although there are no disease-specific findings.

In FPE, moderate anemia (due to iron deficiency) and hypoproteinemia are always present (7, 16, 53). Steatorrhea, sugar malabsorption and deficiency of vitamin K dependent factors may also be observed (39, 41, 51, 54). Although blood in the stool is usually absent, occult blood may be found in about 5% of patients (53, 55). There is usually no peripheral blood eosinophilia and increased total IgE (51).

Specific serology for CD may be necessary to distinguish between FPE and CD in symptomatic infants who have already introduced gluten in their diet (56).

### 8.2 Allergy testing

Allergy testing for food specific IgE is not routinely recommended unless there are associated atopic condition (52).

### 8.3 Other studies

Alpha-1-antitrypsin (A1AT) is a non-dietary serum protein synthesized primarily in the liver. One of the key characteristics of A1AT is its resistance to digestive degradation, meaning that it can survive through the gastrointestinal tract without being broken down by the enzymes in the digestive system. This resistance to digestion makes A1AT a valuable marker for assessing excessive protein losses in the gastrointestinal tract, particularly in conditions such as PLE, including FPE (57). The most reliable method to diagnose enteric protein loss is by measuring the clearance of A1AT from plasma, which is determined by calculating the ratio of 1-day stool quantity to the serum levels of A1AT. Typically, a normal average A1AT clearance is 20 ml/24 h or less (58). Despite its theoretical benefits, such method of measuring A1AT clearance to diagnose enteric protein loss is not commonly used in real-life clinical practice due to several limitations (58).

Xylose, a monopentose sugar, does not require digestion and it is minimally metabolized by the digestive tract. When a standard dose of xylose is ingested, it is absorbed in the upper small intestine and, since it is not metabolized, it largely appears in the urine. The xylose absorption test has historically been a valuable tool in clinical practice for assessing absorptive capacity in the small intestine and distinguishing between absorptive failure and pancreatic digestive failure (59). An oral dose of d-xylose (25 g/500 ml water) is administered, and d-xylose excretion is measured in a 5-h urine collection. Normally, >4 g of d-xylose is excreted in the urine over 5 h (59).

### 8.4 Endoscopy and biopsy

Endoscopy with biopsy is necessary in order to conclusively diagnose FPE. Endoscopic findings are similar to those concerning coeliac disease, only less severe (60).

The diagnosis is confirmed by the presence of intestinal villous atrophy in different degrees and crypt hyperplasia (16, 61) with reduced crypt-villous ratio, a sensitive marker of morphological changes due to jejunal damage (62).

Although less severe, the histology findings are similar to those witnessed in CD (60). Intraepithelial lymphocytes are prominent; conversely, the eosinophils infiltration is inconsistent (63–67). Lymphocytes can also be found in the lamina propria (68), and the amount of mucosal lipid may be increased (69). Columnar cells of the normal jejunum are replaced by crypt cells of a more cuboidal, immature type (5). The epithelial cells bear short microvilli containing large aggregates of lysozymes and abnormal nuclei (70). The basement membrane is thickened. The renewal rate of the epithelial cell increases because of the higher mitotic rate (67, 71). In certain cases, histologic re-evaluation after an elimination diet therapy may be beneficial so as to rule out other diseases (72).

## 9 Differential diagnosis

The differential diagnoses of FPE are listed in Table 1, including a variety of diseases leading to chronic gastrointestinal symptoms with FTT.

**Table 1:** Differential diagnosis of FPE [modified from reference (1)].

	Disease
Allergic	Chronic FPIES Non-EoE EGIDs
Infectious	Viral/bacterial/parasitic gastroenteritis
Gastrointestinal	Celiac disease VEO-IBD Cystic fibrosis
Metabolic	Inborn errors of metabolism Congenital disaccharidase deficiency Type 1 diabetes mellitus
Endocrinologic	Congenital adrenal hypoplasia
Immunologic	Primary immunodeficiency Autoimmune enteropathy
Psychologic	Food aversion Neglect

EGIDs: eosinophilic gastrointestinal diseases; EoE: eosinophilic esophagitis; FPIES: food protein induced enterocolitis syndrome; VEO-IBD: Very early onset inflammatory bowel disease.

Among allergic diseases, the differential diagnosis must be done with non- eosinophilic esophagitis eosinophilic gastrointestinal diseases (non-EoE EGID) and chronic FPIES.

Non-EoE-EGIDs are defined as a condition characterized by gastrointestinal symptoms and eosinophilic infiltration in the gastrointestinal tract, except for the esophagus. Depending on the gastrointestinal segment involved, eosinophilic gastritis, eosinophilic enteritis or and eosinophilic colitis can occur (73).

The clinical manifestation of non-EoE EGID depends on the gastrointestinal tract involved and the depth of involvement (mucosal, muscular, or serous).

In case of mucosal involvement, abdominal pain, vomiting, nausea, diarrhea, malabsorption and protein-losing enteropathy are the main symptoms. Gastrointestinal obstruction is the main symptom in case of muscular involvement. Yet, in case of a serious involvement, the symptoms might be even ascites and peritonitis (74).

According to several laboratory findings, peripheral eosinophilia concerns approximately 70%–80% of patients. An increase in total IgE was reported in 70% of patients, whereas an increase in alpha 2 macroglobulin was found in 92% of cases. The erythrocyte sedimentation rate is normal in most cases. 20%–30% of patients experience an increase in C-reactive protein. Characteristics of malabsorption or protein-losing enteropathy such as iron deficiency anemia and hypoalbuminemia can be found especially in cases of mucosal involvement (74, 75).

Up to date, no gold standard for the diagnosis of non-EoE EGIDs has been set. In 1990, Talley (76) proposed three criteria: the presence of gastrointestinal symptoms, the presence of eosinophils in the gastrointestinal wall or eosinophils in the ascitic fluid and the exclusion of other tissue or peripheral causes of eosinophilia.

Although endoscopic examination may demonstrate non-specific findings (including redness of mucosa, edema, erosion/ulcer, nodules, friability, thickness), biopsies are still needed, as histologic presence of eosinophils is a criterion for diagnosis (76)). However, international consensus regarding the limit value of normal eosinophilic infiltration in the various gastrointestinal tracts has not been reached yet. Moreover, no guidelines on the treatment of non-EoE EGIDs based on the experience and expert opinion are available (75, 74). Spontaneous remission of non-EoE EGID patients occurs in about 40% of cases. The risk of recurrence is higher in patients who have used corticosteroid as initial treatment (74).

Chronic FPIES is reported in infants younger than 4 months of age fed with cow milk (CM) or soy infant formula. Chronic FPIES develops on regular and repeated ingestion of the trigger food and appears as intermittent emesis, watery diarrhea, and FTT. Severe and persistent FPIES can result in dehydration and shock. Infants experiencing chronic FPIES typically regain their normal health status after a 3–10 day transition to a hypoallergenic formula. However, in severe cases, temporary bowel rest and intravenous fluids may be required. Reintroducing the trigger food after a period of avoidance often leads to acute FPIES, for which getting to the correct diagnosis is crucial.

Patients with chronic FPIES show different degrees of anemia, hypoalbuminemia and an increased white blood cell count with a left shift and eosinophilia; in severe cases, metabolic acidosis and methemoglobinemia can also arise (77).

## 10 Management

The cornerstone of the management of FPE is the removal of culprit food from the diet. Based on the severity of symptoms presented and the amount of trigger foods, two types of approaches can be chosen as elimination diets. On one hand, “the bottom-up approach” consists in removing the trigger food only, without eliminating the unsuspected triggers; on the other hand, “the top-down approach” is used in severe cases where FTT and dehydration are the predominant symptoms. With the second approach, a wide variety of foods is excluded. Sometimes it starts with an elemental diet, where the different foods are gradually reintroduced while monitoring the recurrence of symptoms (50, 78). Fundamental to this approach is counselling with a nutritionist to avoid the high risk of nutritional deficiencies (78).

Since CM is the trigger food most frequently implicated in FPE, the international guidelines (79–81) recommend an extensively hydrolyzed (eHF) formula as well as hydrolyzed rice formula (HRF) as first option. The amino acid-based formula (AAF) is recommended as second option, unless the patient has severe FPE with hypoproteinemia and failure to thrive. In that case, the AAF is considered as the first option and the eHF as the second (if the infant refuses the AFF).

Soy-based formulas are considered as first or second option in children older than 6–12 months. It may also be considered in infants who are either refusing or not tolerating an eHF as well as in vegan families (79–81).

In severe clinical manifestations, albumin infusion and treatment of EV corticosteroid may become necessary (15).

For individuals experiencing mild FPE symptoms not suggesting IgE-mediated allergy or FPIES and with negative allergy test results for the suspected trigger food, reintroduction can be safely conducted at home. In case of recurrence of symptoms, the trigger food must be removed again from the diet with a new reintroduction attempt after 6 months (1). No cases of conversion from FPE to FPIES or IgE-mediated food allergy for the same trigger food have been described so far; as well as cases of concomitant IgE hypersensitivity to other food are not reported in literature.

The introduction of weaning foods can follow the usual recommendations without any particular restrictions (1).

## 11 Natural history

Unlike coeliac disease, FPE is usually a transient condition that resolves in most cases by 1–2 years of life (16, 38, 82), although few cases persisting into childhood have also been described (26). In children who were diagnosed at an older age, tolerance developed at an older age, too, although most infants became tolerant by 3 years (10).

A summary of the clinical and laboratory characteristics, the diagnostic criteria and natural history of FPE is shown in Table 2.

**Table 2:** Clinical, laboratory characteristics, diagnostic criteria and natural history of FPE [modified from references (1, 51, 83, 84)].

	FPE	Chronic FPIES	Non-EoE EGIDs
Age of onset	<9 months (typically 1–2 months); can also occur in older children	First weeks-months of life	EoGE: Children <5 years; EoG: older age group
Culprit food			
Most common	Cow milk, soy	Cow milk, soy	Cow milk, wheat
Less common	Wheat, egg, soy		Egg, soy, peanut/tree nut, fish/shellfish
Multiple foods	Rare	Rare	Common
Feeding at onset	Formula	Formula	Varied solid feeding
Clinical manifestations			
	Diarrhea, intermittent vomiting, FTT, steatorrhea, bloody stools (rare)	Intermittent vomiting, diarrhea, FTT	Vomiting, abdominal pain, diarrhea
Co-morbid atopy	20–40%	40–60%	50–70%
Laboratory findings			
	Anemia Hypoalbuminemia Iron deficiency	Anemia Eosinophilia Neutrophilia Methemoglobinemia Metabolic Acidosis	Anemia Iron deficiency Hypoalbuminemia ESR is usually normal
Allergy evaluation			
Food prick skin test Serum food-allergen IgE Total IgE Peripheral blood eosinophilia	Negative Negative Normal Absent	Negative, + in 25% of cases Negative, + in 25% of cases Normal or elevated Present	Negative Negative Elevated or normal Present
Stool studies	Fecal fat	Occult blood PMN Eosinophils Reducing substances	Fecal fat
Urine studies	Low d-Xylose excretion	–	Low d-Xylose excretion
Biopsy findings			
	Variable, patchy villous atrophy Crypt hyperplasi Lymphocytic infiltrate	Friable mucosa Ulceration Villous atrophy Crypt abscesses Inflammatory cell infiltrates	Erosion Ulceration Nodularity Erythema Friability Eosinophilic infiltrates
Diagnosis	Clinical and histological	Confirmatory OFC	Clinical and histological
Treatment	Avoidance of the culprit foods	Avoidance of the culprit foods	Dietary Therapy Glucocorticoids Lirentelimab Cromolyn Ketotifen Montelukast Azathioprine Monoclonal antibodies
Time for improvement	Several weeks (1–2 weeks)	3–10 days	Prednisolone (usually given at an initial dose of 30–40 mg/day) usually induces remission within 2 weeks
OFC	Vomiting and/or diarrhea in 40–72 h	Acute recurrence of symptoms (vomiting in 1–4 h, diarrhea in <24 h)	Not necessary
Natural history	Resolution <1–2 years	Self-limiting; many cases resolve by 3 years of age	Spontaneous remission in 40% of patients

EoG, eosinophilic gastritis; EoGE, eosinophilic gastroenteritis; ESR, Erythrocyte sedimentation rate; FPE, food protein enteropathy; FPIES, food protein induced enterocolitis syndrome; FTT, failure to thrive; h, hours; Non-EoE EGIDs, Non-esophageal eosinophilic gastrointestinal diseases; OFC, oral food challenge; –, not pathological findings.
